# The relationship between grit and selected personality measures in medical students

**DOI:** 10.5116/ijme.5e01.f32d

**Published:** 2020-01-30

**Authors:** Gerald Isenberg, Andrew Brown, Jennifer DeSantis, Jon Veloski, Mohammadreza Hojat

**Affiliations:** 1 Department of Surgery, Sidney Kimmel Medical College at Thomas Jefferson University, Philadelphia, Pennsylvania, USA; 2 Asano-Gonnella Center for Research in Medical Education and Health Care, Sidney Kimmel Medical College at Thomas Jefferson University, Philadelphia, Pennsylvania, USA

**Keywords:** Grit, personality, empathy, medical students

## Abstract

**Objectives:**

To test the hypothesis that scores on a Grit scale are positively associated with personality measures that are conducive to relationship building (Empathy, Self-Esteem, Activity, and Sociability), but inversely associated with personality measures that are detrimental to interpersonal relationships (Neuroticism-Anxiety, Aggression-Hostility, Impulsive Sensation Seeking, and Loneliness).

**Methods:**

Convenient sampling was used that included 241 medical students at Sidney Kimmel Medical College at Thomas Jefferson University who participated in this ex post facto research. Validated instruments were used to measure Grit, Empathy, Self-Esteem, Activity, Sociability, Neuroticism-Anxiety, Aggression-Hostility, Impulsive Sensation Seeking, and Loneliness. Bivariate correlations and multivariate regression were used to examine relationships between scores on the Grit scale and personality measures.

**Results:**

Results of bivariate correlational analyses showed that scores on the Grit scale were positively and significantly (p<0.01) correlated with measures of Self-Esteem (r=0.35), Empathy (r=0.26), and Activity (r=0.17); but negatively and significantly (p<0.01) correlated with measures of Loneliness (r=-0.28), Aggression-Hostility (r=-0.23), Neuroticism-Anxiety (r=-0.22), and Impulsive Sensation Seeking (r=-0.18). Regression analysis indicated that in a multivariate model, higher scores on Self-Esteem and Empathy and lower scores on Aggression-Hostility were uniquely and significantly associated with Grit scores (R=0.43, p<0.01).

**Conclusions:**

Research hypothesis was partially confirmed, suggesting that medical students with higher Grit scores were likely to have higher empathic orientation in patient care and greater Self-Esteem. Conversely, those with higher degrees of Grit displayed lower levels of Aggression-Hostility and Impulsive Sensation Seeking. The Implications of these findings for medical education are discussed.

## Introduction

The personality trait Grit, defined by Duckworth and colleagues,^1^ in 2007 as “perseverance and passion for long-term goals” suggests that Grit can be a potential driving force for success, retention, and well-being in medical students. The initial studies on Grit provided several interesting findings in the general population as well as in high-achieving cohorts such as Ivy League students and West Point cadets. Among adults over 25, higher Grit scores were indicative of less frequent career changes.^1^ In a sample of West Point cadets, higher Grit scores predicted retention during the rigorous first summer of training.^2^ Furthermore, the study by Duckworth  and colleagues^1^ showed that Grit is correlated with educational attainment among Ivy League undergraduates. Higher Grit scores were associated with higher GPAs in college, and interestingly, lower SAT scores.^1^ This suggested that among the study sample, it was Grit, not baseline academic ability that predicted long-term success.^1^

With regards to Grit’s association with personality attributes, Duckworth and colleagues, examined the associations between  Grit and the Big Five personality factors and found a strong correlation with Conscientiousness.^1^^,^^2^ Other findings have shown Grit to have a significant relationship with higher levels of hope,^3^ positive affects and purpose commitment,^4^ self-control, self-regulation, and engagement,^5^ as well as a negative relationship with an orientation to pleasure as a motivational disposition.^6^^,^^7^

The two dimensions of Grit, the perseverance of effort and consistency of interest, have also been examined in their relation to personality attributes and achievement.  In a study of Grit in college students, perseverance of effort was found to be associated with self-esteem, lifelong learning strategies, and general strategies for learning.^8^ In similar studies, perseverance of effort was correlated with academic achievement, college GPA, college satisfaction, and sense of belonging, and was negatively related to intent to change majors.^9^ Perseverance of effort has been associated with self-efficacy and both Grit dimensions were correlated with self-regulated learning strategies and lower procrastination.^10^

Grit has been studied to a lesser extent in the medical profession. Burkhart and colleagues^11^ in 2014 and Salles and colleagues, in 2017^12^ showed that lower Grit scores appeared to be associated with an increased rate of attrition in general surgery residency. Within surgery residents, Grit has been found to predict greater psychological well-being,^12^^,^^13^ and lower depression^12^ and burnout.^13^ A negative correlation between Grit and burnout was also found in a study of UK physicians.^14^ Collectively, these findings provide evidence for Grit’s potential to emerge as a predictor of well-being and academic success within the population of medical students and physicians. Based on the aforementioned findings, it can be speculated that Grit is positively associated with positive personal qualities and negatively associated with negative personality attributes. Testing this speculation is important and timely, given the findings that Grit can predict academic success and achievement of career goals. Also, the scarcity of empirical research on the role of Grit in personality and educational outcomes in medical students indicate a need to explore the correlates of Grit in physicians-in-training further.

We designed this study to test the hypothesis that medical students scores on the Grit Scale are positively associated with positive personality measures such as Empathy, Self-Esteem, Activity, and Sociability that are conducive to relationship building and well-being, but inversely associated with negative personality measures such as Neuroticism-Anxiety, Aggression-Hostility, Impulsive Sensation Seeking, and Loneliness that are detrimental to interpersonal relationship and well-being.

## Methods

### Research design and setting

This ex post facto research was undertaken in a medical school setting.

### Participants

Convenient sampling was used that included 241 medical students with complete data on Grit and other personality measures used in this study who entered Sidney Kimmel Medical College at Thomas Jefferson University in 2013 and 2014 and voluntarily completed personality surveys administered at the beginning of medical school education (online administration) and the Grit Scale in the third year of medical school. The study sample is convenient, representing 46% of the total students in the two classes (n=528). Of the 241 participating students, 49% (n=118) were men and 51% (n=123) were women. The gender composition was not significantly different from that in the population of students in the two classes (50% gender split).

### Instruments

The following instruments were used in this study:

#### The Grit Scale

This instrument, developed by Duckworth and colleagues^1^^,^^2^ included 12 items, each answered on a 5-point Likert-type scale. A sample item is, “Setbacks don’t discourage me”. Evidence in support of its validity and reliability in the general population and college students have been reported.

#### Empathy

We used the Jefferson Scale of Empathy (JSE), a 20-item validated instrument specifically developed to measure empathy in the context of patient care in medical and other health professions students and practitioners. We used the S-version of the JSE, which was developed for administration to medical students.^15^ Evidence in support of the JSE’s validity^16^^-^^20^ and reliability^16^^, ^^20^ has been reported. The JSE instrument has been translated into 54 languages and used in more than 80 countries.^21^ The possible score range is 20 to 140; a higher score on this scale indicates a greater orientation toward empathic engagement inpatient care. The typical Cronbach alpha coefficient for this instrument, which has been reported in many studies, hovers around 0.75.^15^^,^^16^^,^^20^^,^^21^ A sample item on this scale is “It is difficult for a physician to view things from patients’ perspectives”.

#### Self-Esteem

We used an abridged, five-item version of the Rosenberg Self-Esteem Scale,^22^ which is a measure of the self-acceptance aspect of self-esteem.^23^ This abridged scale has been used with medical and other health professions students.^24^^-^^26^ The reliability coefficient of this abridged scale among health professions students have been reported as 0.72.^25^ A higher score on this scale indicates a higher degree of Self-Esteem. A sample item from this scale is “I feel that I am a person of worth, at least on an equal basis with others”.

#### Activity

We used a seven-item scale from the short form of the Zuckerman-Kuhlman Personality Questionnaire  (ZKPQ) that measures a tendency to be active and to prefer challenging work.^27^ The ZKPQ was developed to measure five basic factors of personality that have a strong biological basis.^27^ Evidence in support of the validity and reliability of this scale in male (α =0.67) and female (α =0.72) college students have been reported.^27^ A higher score on this scale indicates a higher degree of preference for challenging work. A sample item from this scale is “I like complicated jobs that require a lot of effort and concentration”.

#### Sociability

We used a seven-item scale from the short form of the ZKPQ to measure sociability.^27^^,^^28^ Evidence in support of the validity and reliability of this scale in male (α =0.78) and female (α =0.79) college students have been reported.^27^ A higher score on this scale indicates a more sociable personality. A sample item from this scale is “I tend to start conversations at parties”.

#### Neuroticism-Anxiety

We used a seven-item scale from the short form of the ZKPQ that measures a tendency to be tense, to worry, to be overly sensitive to criticism, to be easily upset, and to be obsessively indecisive.^27^ Evidence in support of validity and reliability of this scale in male (α=0.70) and female (α=0.72) college students has been reported.^27^ A higher score on this scale indicates a more neurotic personality. A sample item from this scale is “I often worry about things that other people think are important”.

#### Aggression-Hostility

We used a seven-item scale from the short form of the ZKPQ that measures a tendency to express verbal aggression and to show rudeness, thoughtlessness, vengefulness, spitefulness, a quick temper, and impatient behavior.^27^ Evidence in support of the validity and reliability of this scale in male (α=0.66) and female (α=0.67) college students have been reported.^27^ A higher score on this scale indicates a higher degree of aggression and hostility. A sample item from this scale is “If people annoy me, I do not hesitate to tell them so”.

#### Impulsive Sensation Seeking

We used a seven-item scale from the short form of the ZKPQ that measures a tendency to act quickly on impulse without planning, often in response to a need for thrills and excitement, change, and novelty.^27^ Evidence in support of validity and reliability of this scale in male (α =0.62) and female (α =0.71) college students have been reported.^27^ A higher score on this scale indicates a higher degree of impulsiveness and thrill-seeking behavior. A sample item from this scale is “I often do things on impulse”.

#### Loneliness

We used an abridged, five-item version of the UCLA Loneliness Scale, which is a global measure of loneliness experiences.^29^ The abridged version has been used previously with medical and other health professions students^24^^,^^25^ and its psychometric support in medical students has been reported.^26^ The reliability coefficient of the abridged scale among health professions students has been reported as 0.87.^25^ A higher score on this scale indicates a greater experience of loneliness and a lack of satisfaction with social relationships. A sample item from this scale is “I feel isolated from others”.

### Procedures

The study was approved and determined to be exempt by the university’s IRB due to minimum risk factor for participants and provision of strict confidentiality of individual data. Students completed the Jefferson Scale of Empathy, ZKPQ, Self-Esteem, and Loneliness scales as part of the Jefferson Longitudinal Study of Medical Education.^30^^,^^31^ at the beginning of medical school. They completed the Grit Scale for this study in the third year of medical school.

### Statistical analysis

We calculated Pearson correlation coefficients to examine the bivariate relationship between scores on the Grit Scale with those of each personality measure. To examine the unique contribution of each personality measure to the Grit scores, we used multivariate regression analyses in which Grit was considered as the dependent variable and personality measures as the regressors. The Statistical Analysis System software (SAS, Windows Version 9.3) was used for statistical analyses.

## Results

Descriptive statistics (possible and actual score ranges, means, standard deviations, and internal consistency aspects of reliability (Cronbach alpha coefficients) for each scale used in the study are reported in Table 1.

**Table 1:** Means, Standard Deviations, and Cronbach alpha coefficients of grit and personality measures

Variables	Possible Score Ranges	Actual Score Ranges	M(SD)	Cronbach Alpha Coefficients
Grit	1-5	2.17 – 4.67	3.83 (0.46)	0.79
Self-Esteem	5-20	10 - 20	16.9 (2.5)	0.78
Aggression–Hostility	0-7	0 - 7	1.4 (1.5)	0.63
Impulsive Sensation Seeking	0-7	0 - 7	2.1 (1.7)	0.65
Empathy	20-140	74 - 138	115.7 (10.5)	0.83
Activity	0-7	0 - 7	4.7 (1.9)	0.68
Sociability	0-7	0 - 7	4.4 (2.0)	0.76
Loneliness	5-20	5 - 20	9.8 (2.7)	0.80
Neuroticism–Anxiety	0-7	0 - 7	1.9 (1.7)	0.67

The mean Grit score was 3.83 (standard deviation 0.46), with internal consistency reliability (Cronbach alpha) of 0.79.

**Table 2:** Pearson bivariate correlations between grit and selected personal quality measures

Variable	Grit	Self-Esteem	Aggression–Hostility	Impulsive Sensation Seeking	Empathy	Activity	Sociability	Loneliness	Neuroticism-Anxiety
Grit	1.00								
Self-Esteem	0.35^**^	1.00							
Aggression–Hostility	-0.23^**^	-0.10	1.00						
Impulsive Sensation Seeking	-0.18^**^	-0.05	0.28^**^	1.00					
Empathy	0.26^**^	0.25^**^	-0.15^*^	-0.12	1.00				
Activity	0.17^**^	0.17^*^	-0.04	0.10	0.06	1.00			
Sociability	0.11	0.22^**^	-0.05	0.33^**^	0.14^*^	0.15^*^	1.00		
Loneliness	-0.28^**^	-0.60^**^	0.13^*^	0.05	-0.29^**^	-0.18^**^	-0.37^**^	1.00	
Neuroticism–Anxiety	-0.22^**^	-0.52^**^	0.13^*^	0.05	-0.08	-0.13	-0.26^**^	0.46^**^	1.00

^*^p <0.05; ^**^p <0.01

**Table 3:** Summary results of multiple analysis†

Regressors	Standardized Regression Coefficient	Unstandardized Regression Coefficient	Standard Error	*t *- value
Self-Esteem	0.24	0.06	0.02	3.19^**^
Aggression-Hostility	-0.14	-0.04	0.02	-2.30^*^
Impulsive Sensation Seeking	-0.13	-0.04	0.02	-2.00^*^
Empathy	0.14	0.01	0.003	2.14^*^
Activity	0.10	0.03	0.01	1.72
Sociability	0.05	0.01	0.02	0.70
Loneliness	-0.05	-0.01	0.01	-0.69
Neuroticism-Anxiety	-0.04	-0.01	0.02	-0.66
Intercept	0	2.26	0.49	4.66^**^

^*^p <0.05; ^**^p <0.01; ^†^Grit as the dependent variable and personality measures as regressors (adjusted multiple R=0.43, p< 0.01)

These statistics are slightly higher than those reported in a study conducted with a large, nationally representative internet panel sample in the US (Mean=3.58, SD=0.60, Cronbach alpha=0.74).^32^ The internal consistency aspect of reliability (Cronbach alpha coefficients) for each scale used in the study (ranging from 0.63 to 0.83) are in the acceptable range for psychological measures.

Table 2 presents the intercorrelation matrix of measures used in the study. As shown in the table, bivariate correlations between scores on the Grit Scale and three personality attributes were positive and statistically significant (p<0.01) in this order of magnitude: Self-Esteem (r=0.35), Empathy (r=0.26), and Activity (r=0.17). Conversely, negative and statistically significant (p<0.01) bivariate correlations were found between scores on the Grit Scale, and four personality attributes in this order of magnitude: Loneliness (r=-0.28), Aggression-Hostility (r=-0.23), Neuroticism-Anxiety (r=-0.22), and Impulsive Sensation Seeking (r=-0.18).  The pattern of findings is consistent with those reported in a study of an internet panel sample in the US,^32^ in which correlations of 0.18 and -0.22 were found between Grit scores, and sociability (extroversion) and neuroticism, respectively. Summary results of multiple regression analysis are reported in Table 3. Results showed that Self-Esteem (standardized regression coefficient=0.24, p<0.01) and Empathy (standardized regression coefficient=0.14, p <0.05) could uniquely predict higher scores on the Grit Scale, and Aggression-Hostility (standardized regression coefficient=-0.14, p<0.05) could uniquely predict lower scores on the Grit Scale. The adjusted multiple R for the regression model was 0.43 (p <0.01). The coefficient of determination (R^2^) which is an indication of common variance (overlap) between the dependent and independent variables, or between Grit scores and a combination of personality measures used in this study, was 18% (R=0.43, R^2^=0.432 = 0.18, or 18% common variance). No significant association was observed in the multivariate statistical model between Grit and measures of Impulsive Sensation Seeking, Activity, Sociability, Neuroticism-Anxiety, and Loneliness.

The aforementioned findings partially confirmed our research hypothesis that a higher degree of Grit in medical students is associated with higher scores on positive personality attributes, such as Self-Esteem and Empathy, but inversely associated with a negative personality attribute, such as Aggression-Hostility.

## Discussion

The results of correlational analyses (univariate and multivariate regression) presented herein partially confirmed our research hypothesis regarding significant associations between Grit and positive personality attributes, and an inverse association between Grit and negative personal attributes. The magnitude of the bivariate correlations are not large, but it is interesting to note that positive personality attributes which are conducive to relationship building and well-being showed positive associations with the Grit scores, and negative personal attributes which are detrimental to healthy interpersonal relationships and well-being were negatively related to scores of the Grit scale. This provides a first look at the personality profile of what may be a more successful medical student.

There have been many attempts over the years to improve the system of medical education and training with the stated goal of producing better-trained, more satisfied physicians.^33^^,^^34^  Unfortunately, up to 45% of physicians exhibit symptoms of burnout and thus are more likely to leave the profession.^35^^-^^37^

There is a very real societal cost to having dissatisfied doctors and pervasive physician burnout. Over one-third of all currently practicing physicians are expected to retire within the next decade. In the face of a predicted shortage of 61,000 physicians by 2025,^38^ society needs to retain as many currently practicing physicians as possible.  Even earlier in the careers of doctors, attrition from certain medical residencies, such as general surgery residency, can be as high as 18%.^39^ In order to avoid some of the issues associated with physician burnout and medical students and resident attrition, attention should be placed earlier along the path of medical education. In addition to producing more well-trained young doctors, identification of those future doctors who are likely to stay in the field is paramount. A starting point to address this strategy is to establish what personality characteristics are embodied by a successful, satisfied medical student. In this analysis we have begun to create a personality profile of medical students who are passionate to achieve their professional goals.

### Implications

Despite some attempts to look at Grit in the physician population,^11^^-^^14^ this is the first time, to our knowledge, in which personality correlates of Grit has been studied in physicians-in-training. We have previously shown that Empathy scores erode in the third year of medical school as the program shifts from preclinical to clinical phases of medical education when students become more involved in direct patient-care activities.^40^ We need to undertake longitudinal research to explore if a similar pattern of decline in Grit is observed as students progress through medical school. Such findings have important implications for medical education. For example, if we can assess personality attributes that are significantly associated with a student’s well-being and enhancing interpersonal relationships (e.g., Grit, Empathy, Sociability) at a pivotal stage of medical education, these attributes can be cultivated, ultimately leading to greater efforts to achieve professional goals, thus to more academic success and personal well-being, as well as less attrition and burnout in medical students. Ideally, this well-being will stay with the students and continue into their professional lives, resulting in more practicing physicians who are satisfied and also in improving patient-physician relationships. Moving forward, we plan to follow these students as they advance into their careers to examining their choices of specialties and indicators of their clinical competence in postgraduate medical education.

### Study limitations and future research

This study begins to address the aforementioned aims, using a sample of third-year medical students at a single private institution. Generalization of the findings may be limited by selecting a convenience study who voluntarily participated in the study. However, our findings that the study sample represented the population with regard to gender can mitigate this limitation and increase our confidence about the resemblance of the sample with the respective population concerning gender.

Generalization of the findings is always limited when using single-institution research which is the case in our study. The external validity (generalizability) of our findings can be examined in future research by studying multiple institutional research. Expansion of the study beyond studying personality attributes is also desirable. For example, other areas of future research should include examining medical student academic achievements and satisfaction, and exploring associations between Grit, and attrition, burnout, specialty interest, attitudes toward interprofessional collaboration and teamwork, orientation toward lifelong learning, and experiences throughout medical education and practice, as well as methods for cultivating Grit and other markers of success in medical student populations.

### Acknowledgments

We would like to thank Jennifer Wilson for her editorial assistance.

### Conflict of Interest

The authors declare that they have no conflicts of interest.
